# 

**Published:** 1912-07-15

**Authors:** 


### ANNOUNCEMENTS.

Ohio State Dental Society. It may seem rather early to announce the next neetimg of the Ohio State Dental Society but a very unusual move is contemplated and those most interested are exceedingly desirous that this shall be the banner meeting. The next session will be held at the usual time. December 3rd to 5th, 1912, but in Cincinnati instead of in Columbus, the usual place of meeting. The local committee has already engaged the convention hall in the Sinton Hotel, which will supply one of the finest meeting halls in the State, and has begun its preparations on a large scale for a meeting that will draw all dentists to Cincinnati at that time. We are asked to lend our influence to this end, and we are more than pleased to do anything we can to encourage or promote the enterprise. From what we know of the Cincinnati men we predict that it will be a meeting that no progressive man can afford to miss, and every one should begin to plan for it now. You will hear more particulars later.

The National Association of dental faculties will meet at the New Willard Hotel, Washington, D. C., on Friday and Saturday, September 6th and 7th. The Executive Committee will meet at nine o'clock Friday morning, the general meeting opening at ten o'clock the same morning.

George Edwin Hunt, *Sec'y.*

To Clinicians.—The meeting of the National Dental Association this year in Hotel Willard, Washington, D.C., September 10th to the 13th inclusive, will be unique in many features: There will be a "bumper" attendance, because many states have joined "en masse;" three full day and night sessions will be devoted to papers and discussions and the last day, morning and afternoon sessions, to clinics.

It will be the effort of the Clinic Committee to group the various clinical events as they should appear under the different subjects illustrated and as much in sequence as possible, so that the views of the clinicians may be had at glance. We are particularly desirous that you should present some of your original methods, for no matter how trite or trifling the matter may seem to you, it may be of benefit to your less fortunate brother; it is also possible that he may have some method new to you, and it is only by such reciprocal relations that we advance.

An early reply, that your name may appear in the preliminary programme, stating name; address; clinic title; chair or table; what steps you will illustrate; major instruments desired and what type of patient needed, will be appreciated and meet a prompt response.

We expect to have the largest and most select clinic yet given by the N. D. A. Washington is delightful in September Be with us.

Yours fraternally,
C. J. GREVES,
*Chairman for Clinics.*

---

CONGRESS OF HYGIENE AND DEMOGRAPHY.—Last month we gave an extended notice of this meeting to be held in Washington City, September 23-28. We desire to keep this meeting prominently before our readers, because it will have so much of interest to the dental profession.

The National Mouth Hygiene Association is planning to make an exhibit that will worthily present the dental aspect of the subject and is calling on all members of the profession to contribute both material and money for this purpose.

Any one wishing to contribute in either way or in both ways will be gladly welcomed by the officers.

Address,

DR. W. G. EBERSOLE,

800 Schofield Bldg., Cleveland, O.

---

### PRESENT STATUS OF THE TAGGART-BOYNTON SUIT.

DR. M. F. FINLEY,
Washington, D. C. May 21, 1912

DEAR DOCTOR FINLEY:

I am in receipt of your request for a statement as to the status of the Taggart-Boynton case, and also asking for an expression as to my views as to the ultimate outcome of this case.

I am very glad to give you a statement as to the status of this case, but I am afraid any statement as to my views as to what the decision of the Court of Appeals will be, will not carry with it very much weight, in view of the fact that lawyers are inclined to be optimistic in the matter of their own cases.

Whatever my own views may be on this subject, it is absolutely apparent that Dr. Taggart's attorneys have not the slightest expectation of winning this case for their client. It has only been with the greatest difficulty that we have from time to time been able to get them to take any steps in this case. We had to secure an order from the Court compelling them to take their testimony within a limited time, and after the testimony of both sides was closed they made no effort whatever to have the case placed on the calendar for trial. The defense in this case took the most unusual action of having this case placed on the calendar for trial; otherwise the case would never have come to issue. This attitude on the part of the plaintiff upon whom the burden rests of prosecuting the case indicates clearly that the plaintiff desires to postpone a final decision just as far as possible.

The decision of Mr. Justice Clabaugh has no bearing whatever on the status of the case except in so far as it compels us to bear the burden of prosecuting the appeal. Justice Clabaugh frankly stated in deciding this case that in view of the fact that it had come to his knowledge that both sides intended to appeal the case that he did not feel it necessary or incumbent upon him to read the great mass of testimony taken, leaving it to the Court of Appeals to examine into the testimony, as it would have been called upon to do, whatever the decision of the Lower Court may have been. This frank statement on the part of Justice Clabaugh will really result in our favor as it will insure a careful examination of all the testimony in detail by the Court of Appeals, as it will not have the benefit of any criticism or analysis thereof by the lower court.

The trial Judge in the Lower Court did not even attempt to designate which of the claims of the patent were sustained, but simply in a brief statement held the patent to be valid, which of course means that every one of the claims are sustained. No person no matter how prejudiced he may be could for one instant seriously contend that all the claims embraced in this patent are valid. If such should be held to be the case the result would be disastrous to the dental profession, as some of the claims are so broad as to cover almost the entire field.

The evidence in this case shows clearly that at least three dentists of prominence and irreproachable character practice the process for which Dr. Taggart was granted a patent several years prior to the granting of such patent. If the Court of Appeals believes the testimony of these witnesses, and there has been no attempt to impeach their testimony on this point the Court must decide in favor of the defendant.

My associates and myself have carefully considered this matter from every aspect and we have no hesitancy in stating to you that in our opinion a decision from the Court of Appeals in favor of Dr. Boynton is inevitable.

Very truly yours,

FRED B. RHODES.

P. S.—This is a copy of a letter from attorney for defense in Taggart-Boynton suit to Dr. Finley.

NATIONAL DENTAL ASSOCIATION.—The 1912, session of the National Dental Association will be held in Washington, D. C., September 10th to 13th, and all indications are favorable for this being the most important and successful meeting that this association has ever held.

The Local Committee of Arrangements has selected the New Willard as "Headquarters Hotel" and necessary accommodations for the meetings of the General Sessions and Sections, as well as the "All Day Clinic" on the last day, are to be held in the commodious Ball Room on the eleventh floor of this Hotel. This provides the ideal arrangements for a successful meeting, that is, all under one roof.

The re-organization proposition has been receiving most liberal support from the State Societies which have met since the Cleveland meeting when a constitution, along the lines of the American Medical Association, was tentatively adopted. This question will come up at this meeting for final action and every one interested in the perfecting of a representative National Dental Association should be present. You are respectfully requested to remember this meeting when making your vacation arrangements, as this presents an excellent opportunity to attend the meeting of the National Dental Association and visit our National Capitol.

The details of the program are not complete, but the officers and committees have spared no effort in preparing same, and further information will appear in August and September Journals.

ARTHUR R. MELENDY, *President*.
Deaderick Bldg., Knoxville, Tenn.
HOMER C. BROWN, *Recording Secretary*.
185 East State St., Columbus, Ohio.

